# Effects of Botanical Supplementation on Benzene-Induced Hematological, Histopathological, and Molecular Alterations in Rats: Short title: Botanical Supplementation In Benzene-Induced Leukemia Rats

**DOI:** 10.31661/gmj.v15i.4246

**Published:** 2026-07-15

**Authors:** Nzar Ibrahim Yousif, Sarween Sherzad Rasool, Zhala B Taha, Mohammed M. Hussein M. Raouf, Bakhtawar Ziad Omer, Zahraakhan Maaroof Taher, Abdullah Jawdat Zebari, Zeenath Sufderhusain Sheikh, Hero Ismael Mohammed, Shahrokh Mojarradgandoukmolla, Tanya Salam Salih, Usha Rani Kandula, Sherzad Hassan Radha, Abdulrahman Ismael

**Affiliations:** ^1^ Department of Community Health Nursing, Cihan University-Erbil, Erbil, Kurdistan Region, Iraq; ^2^ Department of Laboratory, Unit of Hematopathology and Flow Cytometry, Nanakali Hospital for Blood Diseases and Cancer, Erbil, Iraq; ^3^ Department of Forestry, College of Agricultural Engineering Science, Salahaddin University-Erbil, Kurdistan Region, Iraq; ^4^ Department of Biology, Faculty of Science and Health, Koya University, Koya 44023, Kurdistan Region – F.R. Iraq; ^5^ Department of Biomedical Sciences, Cihan University-Erbil, Erbil, Kurdistan Region, Iraq; ^6^ Department of Medical Biochemical Analysis, Cihan University-Erbil, Erbil, Kurdistan Region, Iraq; ^7^ Department of Anesthesia Technology, Cihan University-Erbil, Erbil, Kurdistan Region, Iraq; ^8^ Department of Biology, College of Education, Salahaddin University-Erbil, Erbil, Kurdistan Region, Iraq; ^9^ Department of Physiotherapy, Cihan University-Erbil, Erbil, Kurdistan Region, Iraq

**Keywords:** Botanical Supplementation, Benzene-Induced Leukemia, Hematological Alterations, Histopathological Changes, Molecular Alterations

## Abstract

**Background:**

Leukemia is a hematological malignancy characterized by uncontrolled proliferation of immature white blood cells, influenced by both genetic and environmental factors. Benzene is a recognized leukemogenic agent associated with hematopoietic toxicity following prolonged exposure. This study investigated the protective effects of Withania somnifera (ashwagandha), Camellia sinensis (green tea), and curcumin against benzene-induced leukemic alterations in rats.

**Materials and Methods:**

Adult male albino rats received intravenous benzene (99.9%) every two days for 3 weeks to induce leukemic changes. Following induction, rats were divided into three groups (n=6/group), 1:a combination-treated group receiving oral Withania somnifera (200 mg/kg), Camellia sinensis extract (250 mg/kg), and curcumin (200 mg/kg) daily for 30 days; 2:a Withania somnifera-treated group receiving 300 mg/kg daily for 30 days; and 3:a control group. Hematological, histopathological, and molecular analyses were performed using blood, bone marrow, spleen, and liver samples.

**Results:**

Both treatment regimens reduced several benzene-induced hematological, histopathological, and molecular alterations. Hematological parameters in treated groups were maintained within near-normal ranges. Histopathological examination demonstrated partial preservation of liver and spleen architecture in both treatment groups, with reduced inflammatory and degenerative changes compared with untreated benzene-exposed rats. Molecular analysis showed restoration of Nrf2 expression and modulation of Caspase-3 expression following treatment, particularly in the combination-treated group. Blast cell counts were reduced in both treatment groups compared with untreated benzene-exposed animals.

**Conclusion:**

Withania somnifera, alone or in combination with curcumin and green tea extract, demonstrated protective effects against benzene-induced hematological, histopathological, and molecular alterations in rats. The combination treatment showed greater improvement in molecular markers associated with oxidative stress and apoptosis. These findings support further investigation of these botanical compounds as potential supportive agents in leukemia-related disorders.

## Introduction

Leukemia is a heterogeneous group of hematological malignancies that originates in blood-forming tissues, particularly the bone marrow and lymphoid organs. It is characterized by the uncontrolled proliferation and accumulation of abnormal white blood cells in the bone marrow and peripheral blood [1]. The term leukemia is derived from the Greek word leukos, meaning white, due to its historical association with abnormal white blood cells. Leukemia is an aggressive disease associated with bone marrow failure, impaired hematopoiesis, tissue infiltration, and reduced survival [2]. Benzene is a well-known hematotoxic and leukemogenic environmental and occupational pollutant. Chronic exposure to benzene has been associated with hematopoietic dysfunction and an increased risk of hematological malignancies, including leukemia [3, 4].

Benzene and its metabolites can damage bone marrow cells and disrupt immune function through alterations in T-cell populations and immune regulation. In acute myeloid leukemia (AML), immature myeloid blast cells accumulate in the bone marrow and peripheral blood, impairing normal immune defense mechanisms [5].

Conventional anticancer therapies are often associated with toxicity and limited selectivity. Therefore, increasing attention has been directed toward natural compounds with antioxidant, anti-inflammatory, and anticancer activities [6]. Ashwagandha (Withania somnifera), commonly known as Indian ginseng, is a medicinal plant with diverse pharmacological properties. Its bioactive constituents, including alkaloids and steroidal compounds, have demonstrated antiviral, antidiabetic, antioxidant, and anticancer activities in experimental studies. In addition, Withania somnifera extracts have shown promising protective effects in tumor cell lines and animal models [7]. Curcumin, the principal polyphenolic compound of Curcuma longa L., has been widely used in traditional medicine, especially in India and China. Previous studies have demonstrated that curcumin can modulate several molecular pathways involved in carcinogenesis and inflammation [8]. These effects include regulation of nuclear factor erythroid 2-related factor 2 (Nrf2), nuclear factor-κB (NF-κB), and inflammatory mediators such as interleukin-6 (IL-6) and signal transducer and activator of transcription-1 (STAT-1) [9].

Green tea, derived from Camellia sinensis, is another widely consumed natural product with recognized health-promoting effects. Recent evidence suggests that green tea polyphenols may contribute to cancer prevention and modulation of chronic diseases. Of particular interest in leukemia research, green tea components have been reported to selectively induce apoptosis in leukemic cells while exerting minimal effects on normal blood cells [10, 11]. Considering the biological activities of these phytochemicals, natural compounds may provide protective effects against benzene-induced toxicity and leukemic alterations. Therefore, the present study investigated the protective potential of Withania somnifera alone and in combination with curcumin and green tea in a rat model of benzene-induced leukemia through hematological, histopathological, and molecular analyses [12].

## Materials and Methods

### Chemicals

The materials used in this study included benzene (99.9% purity; Scharlau, Spain), isopropanol (Chem Lab, USA), Withania somnifera (ashwagandha) extract and green tea extract derived from Camellia sinensis (Now Foods, USA), curcumin extract obtained from Curcuma longa (21st Century Healthcare Inc., USA), methanol (Chem Lab, Belgium), May-Grünwald stain (Chem Lab, Belgium), May-Grünwald-Giemsa (MGG) stain (Atom Scientific, UK), and an automated hematology analyzer (Medonic M51, Sweden). Ketamine hydrochloride (Ketalar®, Pfizer, USA) and xylazine hydrochloride (Xyla®, Interchemie, Netherlands) were used for anesthesia. TTotal RNA extraction kits, reverse transcription kits, and SYBR Green qPCR Master Mix were purchased from Thermo Fisher Scientific (USA).

### Experimental Animals

Adult healthy male albino rats (8-10 weeks old) obtained from the Experimental Animal House of the College of Science, Cihan University-Erbil, Iraq, were used in this study. Animals were maintained under standard laboratory conditions with a 12 h light/dark cycle and were provided with a standard pellet diet and tap water ad libitum. All procedures were conducted in accordance with institutional guidelines for animal care and use to minimize stress and ensure animal welfare. The experimental protocol was reviewed and approved by the Ethics Committee of the College of Science, Cihan University-Erbil, Iraq (CUE-REC/2025/ 11).

### Experimental Design

A total of 32 adult male albino rats weighing 200 ± 20 g were initially included in the study. During the benzene administration period, 8 rats died following injection; therefore, the final experimental sample consisted of 24 rats. The remaining animals were randomly assigned into four experimental groups (n=6/group) using a simple randomization method.

• Group 1 (Positive control group): Rats received intravenous benzene injections every 2 days for 3 weeks and did not receive plant extract treatment [13].

• Group 2 (Negative control group): Rats received a normal diet and served as healthy untreated controls [14].

• Group 3 (Combination-treated group): Rats received intravenous benzene injections every 2 days for 3 weeks to induce leukemia, followed by oral treatment once daily for 1 month with a combination of curcumin derived from Curcuma longa (200 mg/kg), green tea extract from Camellia sinensis (250 mg/kg), and Withania somnifera extract (200 mg/kg) [15, 16].

• Group 4 (Withania somnifera-treated group): Following benzene induction, rats received oral Withania somnifera extract at a dose of 300 mg/kg once daily for 1 month [17].

This experimental grouping allowed comparison among untreated benzene-exposed rats, healthy controls, rats receiving combination therapy, and rats treated with Withania somnifera alone. Administration of Benzene Chromasolv Benzene was used to induce leukemic alterations in experimental rats, while isopropanol served as the solvent for preparation of the dosing solution Each rat received 0.2 mL of freshly prepared benzene solution containing approximately 16 mg benzene per injection, equivalent to approximately 80 mg/kg per administration for a 200 g rat. The solution was prepared using benzene, distilled water, and isopropanol at a 1:5:5 v/v/v ratio and administered intravenously on alternate days for three consecutive weeks. For the weight range 180–220 g, the approximate dose range would be 72.7–88.9 mg/kg per injection. This protocol was used to establish the benzene-induced leukemia model [18]. Following the induction period, benzene-exposed rats were allocated to the designated treatment groups.

### Supplement Preparation

The plant extracts were prepared as aqueous suspensions immediately prior to administration. In the Withania somnifera-treated group, 1800 mg of Withania somnifera powder was suspended in 30 mL of distilled water. In the combination-treated group, 1200 mg of Withania somnifera, 1500 mg of green tea extract derived from Camellia sinensis, and 1200 mg of curcumin extract obtained from Curcuma longa were suspended together in 30 mL of distilled water. The prepared suspensions were homogenized using a vortex mixer at 3000 rpm for 10 min to ensure uniform dispersion of the extracts [7]. The suspensions were maintained under dry conditions at room temperature during the administration period. Fresh suspensions were prepared every 72 h to maintain extract stability and efficacy.

### Supplement Administration

The prepared extracts were administered orally once daily by gavage for 1 month. Rats in the Withania somnifera-treated group received Withania somnifera extract at a dose of 300 mg/kg body weight. Rats in the combination-treated group received a mixture containing Withania somnifera (200 mg/kg), green tea extract from Camellia sinensis (250 mg/kg), and curcumin derived from Curcuma longa (200 mg/kg). All preparations were suspended in 30 mL distilled water, and 1 mL of the corresponding suspension was administered orally to each rat daily. Treatment was initiated after benzene induction to evaluate the protective efficacy of Withania somnifera alone and in combination with curcumin and green tea against benzene-induced toxicity [19].

### Sample Collection

At the end of the experimental period, blood and tissue samples were collected for hematological, histopathological, and molecular analyses. Animals were anesthetized by intramuscular administration of ketamine hydrochloride and xylazine hydrochloride at a dose of 50 mg/kg. Approximately 10 min after anesthesia, blood samples were collected by cardiac puncture using sterile 5 mL syringes and transferred into EDTA tubes for hematological analysis and peripheral blood smear preparation. Following blood collection, animals were euthanized by cervical dislocation, and tissue specimens were carefully excised for further examination [20]. Liver and spleen tissues were dissected using sterile scalpel blades and forceps, while bone marrow samples were collected from the femurs after removal of surrounding soft tissues. All collected tissues were fixed in 10% buffered formalin for histological processing.

### Hematological Parameters

Blood samples collected in EDTA tubes were analyzed within 3 h of collection to preserve cellular integrity and minimize analytical variation. Hematological parameters included red blood cell (RBC) count, hemoglobin (HGB) concentration, hematocrit (HCT), total white blood cell (WBC) count, platelet count, and lymphocyte count. Peripheral blood smears were additionally prepared for blast cell assessment. For smear preparation, one drop of peripheral blood was placed onto a clean glass slide and air dried [21]. The dried smears were fixed in methanol for 10 min, stained with May-Grünwald stain for 20 min, washed with distilled water, and subsequently stained with May-Grünwald-Giemsa stain for an additional 20 min. After staining, slides were washed, air dried, and examined under a light microscope using oil immersion at 100× magnification for blast cell counting and morphological evaluation of blood cells. For blast cell quantification, at least 200 nucleated cells were counted per peripheral blood smear under oil immersion (100× magnification). Blast cells were identified according to standard morphological criteria, including high nuclear-to-cytoplasmic ratio, fine chromatin pattern, prominent nucleoli, and immature cellular morphology. Blast cell counting was independently performed by two blinded investigators who were unaware of experimental group allocation, and the mean values were used for analysis.

### Histological Studies

Following tissue collection, liver, spleen, and bone marrow samples were fixed in 10% buffered formalin to preserve tissue architecture. Tissues were processed using a paraffin tissue-processing system involving dehydration, clearing, and paraffin infiltration. The processed tissues were embedded in paraffin wax blocks for histological examination. Paraffin-embedded tissue blocks were sectioned into approximately 5 µm thick slices using a rotary microtome [22]. Tissue sections were mounted on glass slides, dried, and stained with hematoxylin and eosin (H&E) according to standard histological procedures. Hematoxylin was used for nuclear visualization, whereas eosin stained cytoplasmic and extracellular components. Histological slides were examined under a light microscope by a histopathologist to evaluate tissue architecture and pathological alterations associated with benzene exposure and treatment. Particular attention was given to inflammatory changes, congestion, degeneration, hemorrhage, fibrosis, and disruption of tissue organization.

### Gene Expression by Quantitative Real-Time PCR

Bone marrow samples were collected at euthanasia from all experimental groups (n=6/group), including the control, benzene-treated, benzene + Withania somnifera-treated, and benzene + combination-treated groups. Bone marrow cells were analyzed for gene expression changes associated with oxidative stress and apoptosis [23]. Total RNA was extracted using the GeneJET RNA Purification Kit (Thermo Fisher Scientific, USA) according to the manufacturer’s instructions. Residual genomic DNA contamination was removed using DNase treatment. RNA concentration and purity were assessed spectrophotometrically before reverse transcription. Approximately 1 µg of tTotal RNA was reverse-transcribed into complementary DNA (cDNA) using the High-Capacity cDNA Reverse Transcription Kit (Applied Biosystems, USA) with random hexamer primers [24]. Quantitative real-time PCR (qRT-PCR) was performed using PowerUp™ SYBR™ Green Master Mix (Applied Biosystems, USA) on a StepOnePlus™ Real-Time PCR System (Applied Biosystems, USA). The amplification protocol consisted of initial denaturation at 95 °C for 2 min, followed by 40 cycles of denaturation at 95 °C for 10 s and annealing/extension at 60 °C for 30 s. Melt curve analysis was performed at the end of amplification to confirm specificity of amplification [25].

The primer sequences used in the present study were as follows:

Nrf2

Forward: 5′-TCCAGTCAGAAACCAGTGGAT-3′

Reverse: 5′-GAATGTCTGCGCCAAAAGCTG-3′ 

Caspase-3 (Casp3) 

Forward: 5′-TGTCATCTCGCTCTGGTACG-3′ 

Reverse: 5′-AAATGACCCCTTCATCACCA-3′ 

Gapdh 

Forward: 5′-CAAGTTCAACGGCACAGTCA-3′ 

Reverse: 5′-GGGTCTTACTCCTTGGAGGC-3′ 

All primers were designed to span exon-exon junctions, with amplicon lengths ranging from 80-160 bp and amplification efficiencies between 90-110% (r²≥0.99). Relative gene expression levels were calculated using the ΔCt method: Ct_target − Ct_Gapdh [26].Gapdh was used as the housekeeping reference gene because its expression remained stable across all experimental groups.

### Statistical Analysis

Data are presented as mean ± SD (n=6/group). Statistical analysis was performed using GraphPad Prism version 10.4.1 (GraphPad Software, Boston, MA, USA). Normality of data distribution was assessed using the Shapiro-Wilk test, and homogeneity of variances was evaluated using Levene’s test prior to parametric analysis. Comparisons among experimental groups were performed using one-way analysis of variance (ANOVA) followed by Tukey’s multiple comparison post hoc test. Exact P-values were calculated, and statistical significance was considered at P< 0.05.

### Blinding Procedures

To minimize observer bias, investigators involved in peripheral blood smear blast cell counting and histopathological evaluation were blinded to group allocation during sample analysis. Histopathological slides were coded prior to microscopic examination. Molecular analyses, including RNA extraction and qRT-PCR procedures, were performed using coded samples to reduce analytical bias during gene expression assessment.

## Results

**Figure-1:**
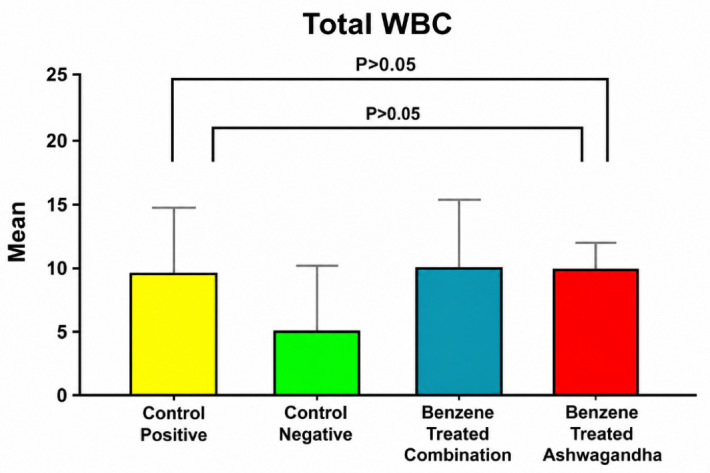
Figure 1. Mean Total White Blood Cell (WBC) Counts in the Experimental Group

**Figure-2:**
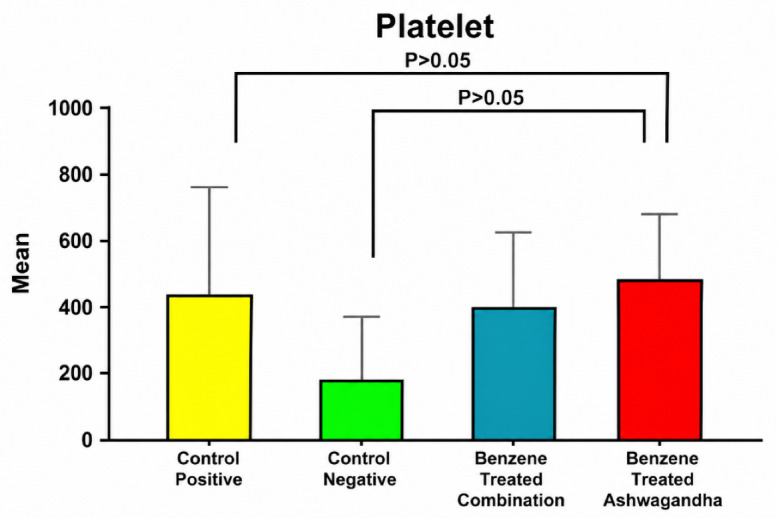
Figure 2. Mean platelet counts in the experimental groups with statistical comparison.

**Figure-3:**
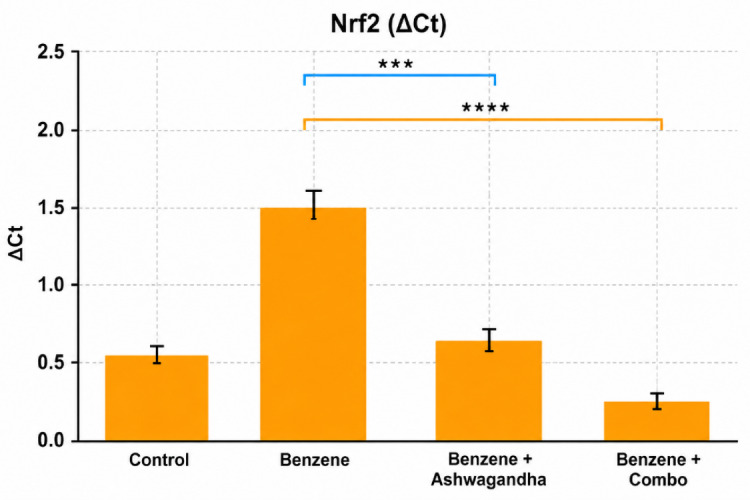
Figure 3. Bone marrow Nrf2 mRNA expression, presented as ΔCt values (mean ± SEM; n = 6/group). Benzene exposure increased ΔCt, indicating reduced Nrf2 expression, whereas Withania somnifera and combined treatment shifted values toward the control level. ***P < 0.001; ****P < 0.0001.

**Figure-4:**
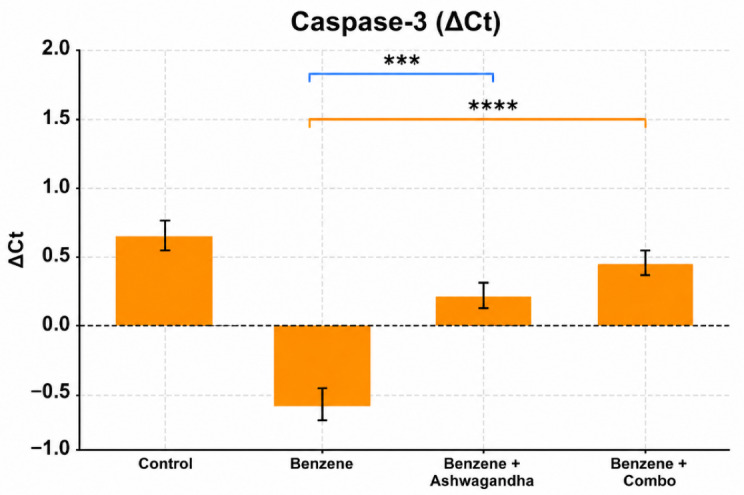
Figure 4. Bone marrow Casp3 mRNA expression, presented as ΔCt values (mean ± SEM; n=6/group). Benzene exposure decreased ΔCt, indicating increased Casp3 expression, while Withania somnifera and combined treatment shifted values toward the control level. ***P < 0.001; ****P < 0.0001.

**Figure-5:**
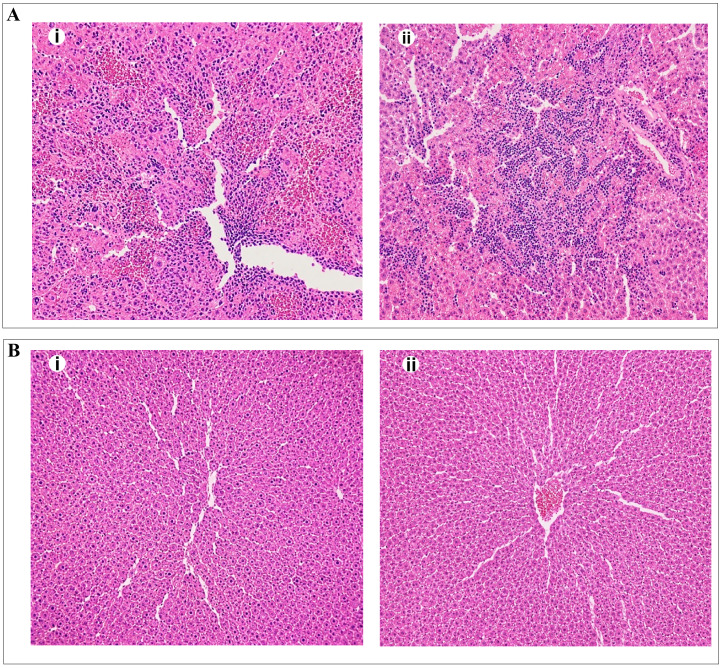
Figure 5. A: Histological sections of the spleen (20×): i) positive-control group showing architectural disruption, congestion, hemosiderin deposition, histiocytosis, sinusoidal dilatation, hemorrhage, and degeneration; ii) combination-treated group showing improved architecture and reduced pathological changes. B: Histological sections of the liver (20×): i) positive-control group showing marked histopathological alterations; ii) combination-treated group showing mild portal lymphocytic infiltration and vascular congestion, indicating partial histological recovery.

### Total White Blood Cell Count

The total white blood cell (WBC) count increased slightly in both benzene-exposed treatment groups compared with the negative control group. The combination-treated group exhibited the highest mean WBC value, while the Withania somnifera-treated group showed a very similar value [27]. The small difference observed between the two treatment groups suggests that both treatment approaches exerted comparable effects on WBC levels. Although the mean WBC counts in treated animals were higher than those of the negative control group, the differences were not statistically significant (P> 0.05). Overall, the findings indicate that Withania somnifera, either alone or in combination with curcumin and green tea, contributed to maintaining WBC levels following benzene exposure.

### Platelet Count

In contrast, platelet counts were higher in both treatment groups compared with the negative control group. The Withania somnifera-treated group exhibited the highest mean platelet count, while the combination-treated group also demonstrated increased platelet levels, with values approaching those observed in the positive control group. This trend suggests that both treatment regimens may have contributed to the restoration of platelet levels following benzene exposure [28]. Despite these numerical differences, statistical analysis revealed that the variations among the groups were not statistically significant (P> 0.05). Therefore, although both treatments appeared to support recovery of platelet counts, no significant differences were detected between the treatment groups or in comparison with the control groups. Gene Expression Gene expression analysis of bone marrow samples demonstrated that benzene exposure markedly altered the transcriptional regulation of genes associated with antioxidant defense and apoptosis. A pronounced increase in the ΔCt value of Nrf2 was observed in the benzene-treated group compared with the control group, indicating reduced gene expression. This finding suggests suppression of antioxidant defense mechanisms following benzene exposure [29]. (Figure-3). In contrast, treatment with Withania somnifera alone or in combination with curcumin and green tea reduced the ΔCt values relative to the benzene-treated group, indicating restoration of Nrf2 expression toward normal levels. The combination-treated group exhibited the greatest improvement, with expression values approaching those of the control group more closely than the Withania somnifera-only group [30]. A similar treatment-related pattern was observed for Casp3 expression. The benzene-treated group showed a marked decrease in the ΔCt value compared with the control group, reflecting increased Casp3 expression and activation of apoptotic pathways in bone marrow cells. Treatment with Withania somnifera partially normalized ΔCt values, whereas the combined treatment consisting of Withania somnifera, curcumin derived from Curcuma longa, and green tea extract from Camellia sinensis produced a more pronounced corrective effect by shifting Caspase-3 expression patterns closer to those of the control group [31] (Figure-4).

Overall, these findings suggest that benzene exposure disrupts the molecular balance between antioxidant defense and apoptosis, while treatment with the investigated plant extracts contributes to restoration of this balance. Among the tested interventions, the combination formulation demonstrated the most substantial protective effect against benzene-induced molecular alterations in bone marrow tissue [32]. Histological Findings Histopathological examination revealed marked alterations in tissue architecture in the benzene-exposed untreated group, whereas partial preservation of tissue structure was observed in the treated groups. In the positive control group, the spleen exhibited pronounced disruption of normal architecture, including secondary follicle formation, congestion of the splenic cords, hemosiderin deposition, sinusoidal dilatation, hemorrhage, and degenerative changes. Liver sections from the same group demonstrated mild mixed inflammatory cell infiltration, predominantly lymphocytes within the portal tracts, accompanied by interface hepatitis, mild vascular congestion, focal fibrosis, and mild fatty changes. These findings indicate substantial tissue injury associated with benzene exposure [33]. (Figure-5 Ai, Bi). In contrast, the combination-treated group demonstrated comparatively milder histopathological abnormalities in both the spleen and liver, suggesting protective and reparative effects of the administered treatment. Splenic tissue showed reduced histiocytic infiltration in the red pulp, decreased hemosiderin deposition, and less severe sinusoidal dilatation, hemorrhage, and degenerative alterations. Similarly, liver sections revealed only mild inflammatory cell infiltration within the portal regions and mild vascular congestion, indicating attenuation of benzene-induced tissue damage following treatment [34]. (Figure-5 Aii, Bii).

## Discussion

Benzene was used in the present study to induce leukemic alterations in male albino rats, and the protective effects of Withania somnifera alone and in combination with curcumin derived from Curcuma longa and green tea extract from Camellia sinensis were evaluated through hematological, histopathological, and molecular analyses.

Overall, both treatment regimens alleviated several benzene-induced toxic effects, although the degree of protection varied according to the evaluated parameter [12].

The combined treatment demonstrated the most pronounced protective activity at the molecular level, whereas both Withania somnifera alone and the combined botanical formulation showed beneficial effects in reducing blast cell counts and preserving tissue integrity.

One of the major findings of the present investigation was the stabilization of total WBCcounts following treatment. WBC values in both benzene-exposed treatment groups remained within near-normal physiological ranges compared with the negative control group. These findings suggest that the botanical treatments may have preserved hematopoietic and immune functions or reduced the suppressive effects of benzene on leukocyte production. The WBC values observed in the combination-treated group were comparable to those reported in studies involving green tea and curcumin supplementation, where WBC levels remained within normal ranges [35]. However, some previous studies reported lower WBC values than those observed in the current work. Such variations are not uncommon and may result from differences in treatment dose, duration of administration, experimental conditions, animal strain, leukemia induction protocol, and phytochemical composition of the administered extracts. Nevertheless, the overall stabilization of WBC counts observed in the present study supports the potential role of Withania somnifera and combined botanical therapy in maintaining leukocyte homeostasis following benzene exposure.

A generally favorable effect was also observed in erythroid parameters. RBC counts remained within relatively similar ranges among the experimental and control groups, indicating that neither treatment regimen resulted in further deterioration of erythrocyte status. RBC values in the Withania somnifera-treated group were consistent with findings from several previous studies, suggesting partial preservation or recovery of erythropoiesis despite benzene exposure. Similarly, the RBC values observed in the combination-treated group were within expected ranges reported for rats receiving plant-based interventions [36].

Hemoglobin (HGB) levels in the present study also indicated a degree of hematological protection, although the recorded values were lower than those reported in some previous investigations involving Withania somnifera supplementation. This discrepancy may reflect incomplete reversal of benzene-induced hematotoxicity, differences in treatment intensity, or variability in the extent of bone marrow injury before treatment initiation. Despite these variations, the overall trend suggests that the botanical treatments contributed to maintaining erythroid parameters within a functional physiological range and may have limited progression toward more severe erythroid abnormalities [37].

A similar interpretation can be applied to hematocrit (HCT) values. HCT levels in both treatment groups were close to those observed in the negative control group, indicating partial stabilization of red cell mass following benzene exposure. HCT values in the Withania somnifera-treated group were comparable to those reported in several previous studies, although considerably higher values have also been documented. Such variability is expected because hematocrit values are influenced by several experimental and physiological factors, including hydration status, duration of treatment, severity of toxic insult, and bone marrow responsiveness. Nonetheless, the findings of the present study indicate that both treatment approaches maintained HCT values within a relatively stable range, with the combination-treated group exhibiting slightly higher mean values than the Withania somnifera-only group.

The lymphocyte findings further support the potential immunomodulatory properties of the tested supplements in benzene-exposed rats. The lymphocyte values observed in the combination-treated group were similar to those reported in studies involving separate administration of green tea and curcumin. This similarity may indicate that the combined regimen preserved immunoregulatory functions associated with its individual phytochemical components. In contrast, the Withania somnifera-treated group exhibited lymphocyte values lower than those reported in some previous investigations, while other studies demonstrated considerably higher values, again reflecting inter-study variability. Nevertheless, the overall pattern suggests that the botanical treatments may help preserve lymphocyte populations and reduce the degree of immune disruption induced by benzene exposure. Considering that benzene and its metabolites are known to adversely affect immune cell populations, these findings are biologically plausible and consistent with the reported antioxidant and immunomodulatory activities of Withania somnifera, curcumin, and green tea phytochemicals.

Among the hematological findings, blast cell counts represented one of the most important indicators of treatment response in the present leukemia model. Blast cell percentages were low in both treatment groups, and no statistically significant differences were observed between them. These findings indicate substantial suppression of leukemic progression following treatment. Importantly, blast cell percentages in the Withania somnifera-treated and combination-treated groups were lower than those reported in several previous studies involving treated leukemic animals with persistent blast cell populations. The reductions in blast cell counts observed in the present study may reflect improvement of the bone marrow microenvironment, attenuation of oxidative damage, suppression of aberrant proliferative signaling, and enhanced elimination of malignant precursor cells through apoptosis-related pathways. These observations suggest that the tested botanical treatments, particularly Withania somnifera combined with curcumin and green tea, may provide anti-leukemic protection against benzene-induced disease progression [38].

Although platelet count differences were not statistically significant, both treatment groups exhibited higher platelet counts than the negative control group, with the Withania somnifera-treated group demonstrating the highest values. Since platelet production is closely linked to bone marrow function, these findings may indicate partial recovery of marrow activity following botanical treatment [27]. While the observed improvements did not reach statistical significance, the upward trend in platelet counts is noteworthy when considered alongside the improvements in other hematological, histopathological, and molecular parameters.

The hematological findings were strongly supported by the molecular analyses. The benzene-treated group demonstrated a marked increase in Nrf2 ΔCt values, indicating suppression of this major antioxidant regulatory gene and suggesting impairment of antioxidant defense mechanisms in bone marrow tissue. In contrast, treatment with Withania somnifera alone, and particularly in combination with curcumin and green tea, reduced Nrf2 ΔCt values toward control levels, indicating restoration of antioxidant gene expression. Restoration of Nrf2 activity is especially important because this transcription factor plays a central role in cellular protection against oxidative stress and toxic injury [39]. The superior response observed in the combination-treated group may reflect synergistic or additive antioxidant effects among curcumin, green tea catechins, and Withania somnifera phytochemicals.

Analysis of Casp3 expression further clarified the molecular basis of these protective effects. Benzene exposure markedly reduced ΔCt values, reflecting increased Casp3 expression and activation of apoptotic signaling pathways in bone marrow cells. This observation is consistent with the well-established cytotoxic and pro-oxidant effects of benzene on hematopoietic tissues. Treatment with Withania somnifera partially reversed these molecular alterations, whereas the combined treatment shifted gene expression patterns more prominently toward those of the control group. These findings suggest that the botanical treatments, particularly the combined regimen, attenuated benzene-induced apoptotic activation in bone marrow tissues. Together with the Nrf2 findings, the molecular results indicate that the tested extracts were effective not only in improving conventional hematological indices but also in restoring the balance between antioxidant defense and apoptosis at the transcriptional level [40].

The histopathological observations were consistent with the hematological and molecular findings. In the untreated benzene-exposed group, the spleen exhibited severe disruption of normal architecture, including follicular disturbances, congestion, hemosiderin deposition, hemorrhage, and degenerative changes. Liver sections from the same group demonstrated inflammatory infiltration, vascular congestion, focal fibrosis, interface hepatitis, and fatty degeneration [41]. These findings confirm that benzene toxicity extends beyond peripheral hematological abnormalities and produces substantial structural injury in hematopoietic and related organs.

In contrast, the spleen and liver tissues of the combination-treated group exhibited considerably milder lesions, including fewer histiocytes, reduced hemosiderin deposition, and less severe congestion, hemorrhage, and degeneration. Similarly, liver sections from treated animals displayed only mild inflammatory and vascular alterations [42]. These findings indicate that the botanical treatments exerted protective and reparative effects on tissue morphology and reduced structural damage associated with benzene-induced leukemia.

Collectively, the findings of the present study suggest that Withania somnifera, curcumin derived from Curcuma longa, and green tea extract from Camellia sinensis exert protective effects against benzene-induced leukemic and hematotoxic alterations through several complementary mechanisms [43]. These mechanisms likely include antioxidant activity, modulation of immune responses, maintenance of hematopoietic homeostasis, attenuation of tissue injury, and regulation of genes associated with oxidative stress and apoptosis. The superior molecular response observed in the combination-treated group suggests that phytochemicals with overlapping but distinct biological activities may provide broader protection than single-agent therapy alone. Nevertheless, the favorable effects observed with Withania somnifera alone also demonstrate its intrinsic anti-leukemic and cytoprotective potential.

Despite these promising findings, several limitations should be acknowledged. The sample size was relatively small, and mortality during benzene induction reduced the number of animals available for final analysis. In addition, only selected hematological, histopathological, and molecular parameters were evaluated. Future investigations should therefore include larger sample sizes, longer treatment durations, dose-response studies, and broader molecular profiling of pathways involved in oxidative stress, apoptosis, inflammation, and hematopoietic regulation. Further studies assessing bone marrow cellularity, immunophenotypic alterations, and additional biomarkers of oxidative and inflammatory injury would provide deeper insight into the mechanisms underlying the protective effects of these botanical compounds. The study demonstrated that Withania somnifera alone and in combination with curcumin and green tea reduced several hematological, molecular, and histopathological disturbances induced by benzene exposure in rats [44, 45]. The combination treatment produced the greatest improvement in Nrf2 and Casp3 expression patterns, along with favorable hematological outcomes and reduced tissue damage. These findings highlight the potential of these botanical agents, particularly in combination, as protective or adjunct therapeutic candidates for experimental leukemia-related disorders.

## Conclusion

The benzene exposure caused significant hematological, molecular, and histopathological alterations in rats, whereas treatment with Withania somnifera alone or combined with curcumin from Curcuma longa and green tea extract from Camellia sinensis reduced these toxic effects. Both treatments improved blood parameters, reduced blast cell counts, preserved tissue structure, and modulated Nrf2 and Casp3 expression, indicating restoration of antioxidant balance and reduced apoptosis. The combination treatment showed greater protective efficacy, particularly at the molecular level, suggesting synergistic effects among the botanical compounds. These findings support the potential use of these natural supplements as supportive agents against benzene-induced leukemic damage, although further studies with larger sample sizes and broader molecular analyses are needed.

## Conflict of Interest

The authors declare that there are no conflicts of interest regarding the publication of this study.

## AI Disclosure Statement

During the preparation of this manuscript, AI–based language assistance (ChatGPT) was used to support manuscript restructuring, language polishing, grammar correction, and improvement of clarity and flow. The authors reviewed, edited, and approved all AI-assisted content and take full responsibility for the accuracy, integrity, and final version of the manuscript.
